# Echocardiographic diagnosis of the different phenotypes of hypertrophic cardiomyopathy

**DOI:** 10.1186/s12947-016-0072-5

**Published:** 2016-08-12

**Authors:** Vito Maurizio Parato, Valeria Antoncecchi, Fabiola Sozzi, Stefania Marazia, Annapaola Zito, Maria Maiello, Pasquale Palmiero, on behalf of Italian Chapter of ISCU

**Affiliations:** 1 Cardiology Unit and EchoLab of Emergency Department, Madonna del Soccorso Hospital, Politecnica delle Marche University, 3-7, Via Manara, San Benedetto del Tronto-Ascoli Piceno, 63074 Italy; 2 Cardiology Unit, Sarcone Hospital, Terlizzi, Bari Italy; 3 Cardiology Unit, University Policlinico Hospital, Milan, Italy; 4 Cardiology Unit, Giannuzzi Hospital, Manduria, Italy; 5 Cardiovascular Diseases Section, Department of Emergency and Organ Transplantation (DETO), University of Bari, Bari, Italy; 6 ASL BR, Health Center, Districtual Cardiology, Brindisi, Italy

**Keywords:** Hypertrophy, Cardiomiopathy, Echocardiography

## Abstract

Hypertrophic Cardiomyopathy (HCM) is an inherited cardiovascular disorder of great genetic heterogeneity and has a prevalence of 0.1 – 0.2 % in the general population. Several hundred mutations in more than 27 genes, most of which encode sarcomeric structures, are associated with the HCM phenotype. Then, HCM is an extremely heterogeneous disease and several phenotypes have been described over the years. Originally only two phenotypes were considered, a more common, obstructive type (HOCM, 70 %) and a less common, non-obstructive type (HNCM, 30 %) (Maron BJ, et al. Am J Cardiol 48:418 –28, 1981). Wigle et al. (Circ 92:1680–92, 1995) considered three types of functional phenotypes: subaortic obstruction, midventricular obstruction and cavity obliteration. A leader american working group suggested that HCM should be defined genetically and not morphologically (Maron BJ, et al. Circ 113:1807–16, 2006). The European Society of Cardiology Working Group on Myocardial and Pericardial Diseases recommended otherwise a morphological classification (Elliott P, et al. Eur Heart J 29:270–6, 2008). Echocardiography is still the principal tool for the diagnosis, prognosis and clinical management of HCM. It is well known that the echocardiographic picture may have a clinical and prognostic impact. For this reason, in this article, we summarize the state of the art regarding the echocardiographic pattern of the HCM phenotypes and its impact on clinical course and prognosis.

## Background

Hypertrophic Cardiomyopathy (HCM) is an inherited cardiovascular disease and its prevalence is estimated to be one case per 500–1000 among the general population.

Hundred mutations in more than 27 genes are associated with the HCM phenotype; most of them encode for sarcomeric structures, while only 5–10 % of HCM patients show other genetic mutations or non genetic causes [1].

For this reason HCM can be mainly meant as a sarcomeric disease, with myocardial fibers disarray as its histological hallmark.

In 2006, the American Heart Association Working Group [2] suggested that HCM should be defined genetically and not morphologically.

Subsequently, the European Society of Cardiology Working Group on Myocardial and Pericardial Diseases recommended a morphological classification [3] including non- sarcomeric forms of HCM. The key point of this latter approach is that clinical evaluation of patients more often starts with the finding of a hypertrophied heart rather than a genetic mutation.

For these reasons, in this article, we review the echocardiographic pattern of the principal HCM phenotypes.

## Differential diagnosis of cardiac hypertrophy

### Several heart diseases may present with hypertrophy

Rapezzi et al. [4] recently published a review article summarizing how clinical, electrocardiographic and echocardiographic features can suggest, in this setting, a specific aetiology for hypertrophy.

Metabolic disorders and congenital syndromes are usually diagnosed very early in lifetime but some types of amyloidosis and Anderson-Fabry disease are frequently discovered in adulthood and cardiac hypertrophy sometimes could be the first clue.

Amyloidosis is often suggested by the presence of pericardial effusion and a ground-glass appearance of myocardium with the involvement of both ventricular chambers, interatrial septum and AV valves tissue.

Storage and infiltrative diseases (e.g. Anderson-Fabry, Danon and Pompe diseases) are commonly associated with severe concentric LVH. In Noonan Syndrome the obstruction of right ventricular outflow can be detected.

For these reasons it is very important to make a correct differential diagnosis between HCM and other heart diseases presenting with hypertrophy.

## The HCM diagnosis

HCM diagnosis is based on the presence of hypertrophied left ventricle in the absence of other disorders that could be responsible for it, such as pressure overload diseases (mainly arterial hypertension and aortic valve stenosis).

ECG is an essential tool to make a suspicion of HCM. In 75 % to 95 % of HCM patients the ECG shows changes in the form of left ventricular hypertrophy [5]. Twenty-five percent of patients exhibit a left anterior hemiblock or a left bundle branch block [5]. The configuration of hypervoltage and giant negative T waves is typical for apical forms, and pseudoinfarct Q waves are typical for obstructive forms [5]. Peripheral low voltage suggests a storage disease or cardiac amyloidosis [4]. A normal ECG does not exclude the presence of HCM but can suggest a mild manifestation of the disease.

Even if cardiac magnetic resonance (CMR) ability, in the assessment of HCM, is improving [6], especially for intra-myocardial fibrous tissue or scar detection using delayed-enhancement imaging, echocardiography remains the principal tool for the diagnosis and morphological characterization of HCM.

## Echocardiographic evaluation

It is well known that the M-mode or 2D cut-off value of left ventricular wall thickness to make a diagnosis of HCM is:

- ≥15 mm in adults;
- >12–15 mm in relatives;
- ≥2 Standard Deviation greater than the Body-Surface-related normal values in pediatric patients [7].

The HCM diagnosis requires the absence of other cardiac or systemic diseases susceptible to producing a similar degree of hypertrophy [8].

All ventricular walls should be analysed at multiple levels but measurements have to be done in end-diastole [9], preferably in short axis view [1].

In 1995, Klues HG [8] said that in hypertrophic cardiomyopathy, the distribution of left ventricular hypertrophy is characteristically asymmetric and particularly heterogeneous, encompassing most possible patterns of wall thickening, from extensive and diffuse to mild and segmental, and with no single morphologic expression considered typical or classic. A greater extent of left ventricular hypertrophy is associated with younger age.

The greatest wall thickness measured at any site in the LV chamber at end diastole is regarded as the maximal wall thickness and a marker of the magnitude of LV hypertrophy. Maron MS et al. [10] found a non-linear and parabolic relation between greater LV wall thickness and NYHA class. Therefore, marked symptoms were most commonly associated with moderate degrees of LV hypertrophy (wall thickness of 16 to 24 mm) but less frequently with extreme hypertrophy (>30 mm) or mild hypertrophy (<15 mm).

Beyond the accurate evaluation of hypertrophy distribution and entity, ultrasounds allow the characterization of left ventricle (LV) systolic and diastolic function, left atrium (LA) volume, left ventricle outflow tract (LVOT), right ventricle outflow tract (RVOT), mid-ventricular obstruction (MVO), apical morphology, mitral valve (MV) + systolic anterior movement (SAM) and pulmonary pressure.

Although a genetic-echocardiographic pattern relationship has not been confirmed [11], according to some studies [12–14], the septum contours could suggest specific HCM genotypes. In particular a reverse curvature was found to be predictive of MYH7/myofilament mutations [14].

### Several new echo-techniques have been applied to HCM

An hypertrophy confined to the apex or to the anterolateral wall could be missed and sometimes the use of contrast agents for cavity opacification is necessary, as like as for the detection of apical aneurysms and clots [15, 16, 17].

Three-dimensional echocardiography (3DE) is supposed to be more accurate in the mass quantification but there are still few data about its routine use in clinical practice [15, 17].

Strain rate imaging, obtained either by Tissue Doppler Imaging (TDI) and Speckle Tracking Echocardiography (STE), is emerging as a useful tool to differentiate HCM from hypertensive cardiomyopathy since more remarkable reductions in strains were demonstrated in HCM patients comparing to the other [18, 19].

Longitudinal strain analysis by STE enables early detection of left ventricular (LV) contraction abnormalities in patients with preserved ejection fraction. Yang H. et al. [20] found that patients with HCM have abnormalities in myocardial mechanics that are related to the site of abnormal myocardial hypertrophy. They showed that apical HCM and septal HCM have common mechanical abnormalities. Longitudinal strain is lower, circumferential strain is higher, and twist is apically displaced. The extent of these abnormalities and their regional expressions vary according to the degree of hypertrophy in every segment. However, some abnormalities are present even in segments with relatively normal wall thickness, likely because of underlying disarray or fibrosis in segments without marked thickening. These findings validate the concept that abnormalities in function are related to the site and degree of hypertrophy.

In Maron’s classification phenotypes [21], by using global longitudinal strain (GLS), Reant P. et al. [22] demostrated that a lower GLS values correlate with several prognostic markers (higher LV mass, higher LV filling pressures, abnormal blood pressure response during exercise test), reflect a more intrinsic myocyte dysfunction than other markers and allow earlier detection of LV systolic function abnormalities, while EF is usually preserved in HCM. They demonstrated also that type III pattern of Maron’s classification [20] (septum + at least a part of LV free wall) exhibits a worse profile than other patterns, with a significantly lower GLS values.

At the moment there are not reproducible data to provide specific cut-off for strain measures in HCM patients [15].

LV untwisting, assessed by speckle tracking echocardiography (STE), may be a novel parameter for evaluating LV relaxation. Van Dalen B. et al. [23] found delayed untwisting to be a rather uniform characteristic of patients with HCM regardless of the extent and site of LV hypertrophy, which is in agreement with the results of a study published by Spirito and Maron [24]. But they found also an important influence of the pattern of hypertrophy on LV twist in HCM, which provides further insight into the pathophysiology of this disease [23].

Potential misdiagnosis may also occur in athletes’ left ventricle hypertrophy (LVH). In these cases the distinction between physiological and pathological hypertrophy has important consequences for the participation in strenuous physical activities.

Differential features include LV cavity dilation in athlete’s heart and the presence of LA enlargement in HCM [5]. HCM patients still have impaired systolic and diastolic function on Tissue Doppler Imaging (TDI) analysis, whereas athletes typically demonstrate normal or supranormal TDI velocities. Finally athlete’s hypertrophy tends to revert stopping training for some months.

Echocardiography is also important for patients’ follow-up, prognostic evaluation [5] and therapeutic management since Trans-Thoracic Echocardiography (TTE) or Trans-Esophageal Echocardiography (TEE) are recommended to guide alcohol septal ablation and surgical myectomy procedures [15].

Finally echocardiography is fundamental in the clinical screening of HCM patients’ relatives [1].

The 2008 ESC-Guidelines on Stress-Echocardiography, published by Sicari R [25], recommended the use of dypiridamole test in HCM patients in order to evaluate the coronary flow reserve, using PW-doppler on LAD coronary artery.

However, since 2009 Maron MS [6] supported an emerging role for CMR in the contemporary evaluation of patients with HCM.

In this article we review the state of the art of the HCM echocardiographic diagnosis focusing on the echocardiographic patterns of the more common phenotypes.

#### Left ventricle diastolic dysfunction

Abnormalities of diastolic function can be observed in about 80 % of patients with HCM, regardless of the morphological phenotype [15]. The diastolic dysfunction is a physio-pathological aspect of great value in HCM patients, both for the earliness of the onset, for the explanation of the severity of symptoms and for informations on prognosis.

The LV diastolic dysfunction is the result of regional diastolic abnormalities of variable magnitude, and it is accentuated by an asynchrony of relaxation. Its degree appears poorly correlated with the extent of hypertrophy. Alterations can affect both early and end phase of the diastole.

Several parameters have been validated to study the diastolic function. Among them: mitral flow doppler analysis, tissue doppler velocities, left atrium size and function. Simple and repeatable indices are represented by the iso-volumetric relaxation time (IRT), usually elongated, and the deceleration time (DT) of E-diastolic wave. The analysis of the pulmonary venous flow doppler pattern provides additional data that can be interpreted and become useful in the clinical management of the patient, since the atrial reversal velocity and its duration have a significant correlation with LV end-diastolic pressure [26].

Following the Finocchiaro et al. recommendations [27], in HCM patients LV filling must be assessed by pulsed doppler at the level of the mitral opening tips. The pattern of LV filling is classified as follows. Restrictive filling pattern: in the presence of E-deceleration time <120 ms or of E/A wave **≥** 2 associated with E-deceleration time **≤** 150 ms. Abnormal relaxation: E/A <1 associated with E-deceleration time >220 ms. Normal (or ‘pseudonormal’): intermediate filling pattern. It should be measured the peak of myocardial early diastolic velocity at the lateral mitral annulus (lateral E’) and transmitral to tissue doppler imaging (TDI) early diastolic velocity ratio (E/E’; using tissue Doppler imaging). The LA and right atrial (RA) volumes must be measured in systole just before the mitral valve opening, using a monoplane area-length method. According to the ASE guidelines, diastolic dysfunction is defined in the presence of severe LA dilation [indexed left atrial volume (LAVi) > 40 mL/m2], increased E/E’ (>15), reduced E’ velocity (<8 cm/s) and a restrictive pattern [15].

Diastolic dysfunction equally affects patients with HCM regardeless of the distribution of hypertrophy and it’s associated with various clinical and echocardiographic variables such as LV obstruction [27].

Diastolic dysfunction is a large contributor to the HCM patho-physiology and it is a major trait of the disease [27].

The distribution of the ventricular and septal wall thickening in HCM varies widely. Ventricular hypertrophy can be focal or diffuse, asymmetrical or concentric, obstructive or non-obstructive.

In HCM, diastolic dysfunction is independent from the morphological pattern. The main correlates of diastolic dysfunction are LV obstruction, age, degree of hypertrophy and mitral regurgitation [28].

Some studies have noted a statistical significance correlations between E/e’ ratio and LV filling pressures. This is present in a large range of annular velocities, including patients with a lateral annular e’ velocity >8 cm/sec [26]. But a recent study conducted by Geske JB et al. [29] noted a modest correlation in patients with HCM between severely impaired LV relaxation and markedly reduced annular velocities. Other clinical researches show that the E/e’ ratio correlates with exercise tolerance in adults [30] and in children [31] with HCM. In addition, septal e’ velocity appears to be an independent predictor of death and ventricular dysrhythmia in children with HCM [31].

LA size and more accurately its volume, provide important prognostic information in HCM [32, 33]. LA enlargement in HCM has multifactorial origins: the severity of mitral regurgitation, the presence of diastolic dysfunction and possibly atrial myopathy [15]. The assessment of LA function via Doppler echocardiographic techniques has been performed by indirect methods using mitral flow and pulmonary venous inflow signals and LA volumes using 2D and 3D echocardiography during the different atrial phases [26, 32–34].

Other indirect measures of LA function have included the calculation of LA ejection force and kinetic energy, which are increased in patients with obstructive HCM and are reduced (though not normalized) after relief of obstruction [35]. Strain imaging of the LA allows for more direct assessment of LA function. Longitudinal strain of the LA by tissue Doppler and 2D speckle-tracking during all three atrial phases was assessed in HCM. LA strain values are reduced in patients with HCM compared with those with secondary LV hypertrophy [36].

## Phenotypes classification

HCM is an extremely heterogeneous disease and several phenotypes have been described over the years [37–39].

Originally only two phenotypes of HCM were considered: a more common, obstructive type (HOCM, 70 %) and a less common, non-obstructive type (HNCM, 30 %) [37, 40].

**Fig. 1:**
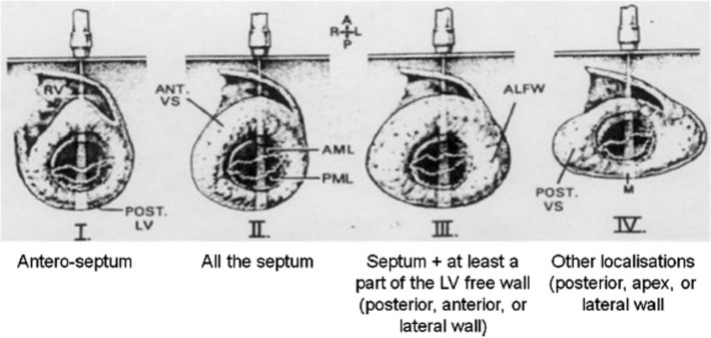
The four phenotypes of Maron’s classification (1981) (from reference 21)

In 1981, Maron BJ [21] published a four types classification. Type I: hypertrophy involving the basal septum; type II: hypertrophy involving the whole septum; type III: hypertrophy involving septum, anterior, and anterolateral walls; type IV: LV apical hypertrophy (Fig. 1).

Nowadays, this classification, based on hypertrophy distribution, is probably the most popular [21].

In 1995 Wigle ED et al., after a long debate, [37] considered three types of functional phenotype: subaortic obstruction, midventricular obstruction and cavity obliteration [41].

Syed IA et al. [42] considered at least five major anatomic subsets based on the septal contour, as well as the location and extent of hypertrophy: reverse curvature, sigmoidal septum, neutral contour, apical form, mid-ventricular form.

*Reverse curvature septum HCM* shows a predominant mid-septal convexity toward the left ventricular (LV) cavity with the cavity itself often having an overall crescent shape. Dynamic subaortic obstruction may be present in this form usually with systolic anterior motion (SAM) of the mitral leaflets and turbulent flow in the outflow tract.

*Sigmoid septum HCM* shows a generally ovoid LV cavity with the septum being concave to the LV cavity and a prominent basal septal bulge. Subaortic obstruction is present in this form usually with SAM of the mitral leaflets and a posteriorly directed jet of mitral regurgitation.

*Neutral septum HCM* shows an overall straight septum that is neither predominantly convex nor concave toward the LV cavity. Subaortic obstruction is less present.

*Apical HCM* shows a predominant apical distribution of hypertrophy. Myocardial delayed enhancement is seen in the LV apex at the site of maximal hypertrophy in this example.

*Mid-ventricular HCM* shows predominant hypertrophy at the mid-ventricular level. In this form a thinned and dyskinetic apical pouch is also present. Obstruction is at the level of the papillary muscles. No mitral SAM. Myocardial delayed enhancement may be seen in the dyskinetic apical pouch.

The most common HCM morphology is reverse curvature and it is most associated with identifiable HCM-associated gene mutations [42].

Recently, Helmy SM [39] proposed a classification including four different patterns which show a good correlation with clinical and ecg presentation (Table 1).

**Table 1:** Helmy’s four-patterns classification. (Modified from ref. 38)

	Distribution	Clinical features
Pattern 1	Septal hypertrophy alone	Less symptomatic phenotype
Pattern 2	Septum and adjacent segments’ hypertrophy but not apical hypertrophy	Less symptomatic phenotype
Pattern 3	Apical in combination with other LV segments’ hypertrophy	More easily detectable with the ecg
Pattern 4	Apical hypertrophy alone	More easily detectable with the ecg

Considering these classifications, we summarize the echocardiographic features of the most common phenotypes.

## Echocardiographic pattern of principal phenotypes

### Asymmetric septal hypertrophy

Most patient with diagnosis of HCM have an asymmetric septal hypertrophy (ASH) with or without subaortic obstruction. For this reason it is considered the most common phenotype.

**Fig. 2:**
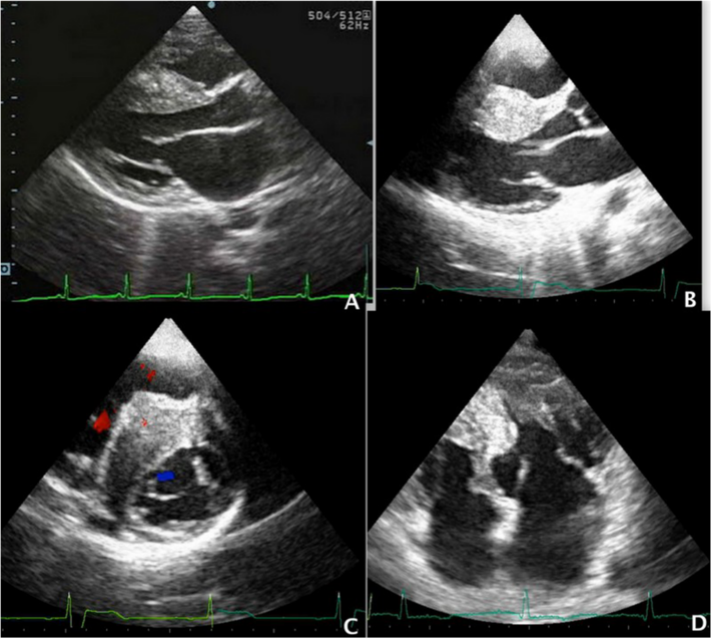
**a** PLAX view demonstrating the asymmetrical hypertrophy of the interventricular septum over the posterior wall with a ratio >1.3. **b** Massive septal hypertrophy characterized by a septal diastolic thickness > 30 mm. **c** Massive septal hypertrophy with RVOT obstruction by the projection of the massively hypertrophied interventricular septum into the right outflow tract. **d** MOHC with the ‘hourglass’ shaped left ventricle consisting of two different chambers: the proximal and the distal chamber

The diagnosis is defined by a septal-to-posterior diastolic wall thickness ratio ≥ 1.3 [9] (or ≥1.5 in hypertensive patients) (Fig. 2A).

It corresponds to reverse curvature and sigmoid septum of Syed’s classification [50].

False positives may be due to: 1) the presence of a right ventricular moderator band or LV tendon that may result in overestimation of septal thickness; 2) the presence of a sigmoid septum in an elderly patient (often inaccurately reported as ASH) which may be also associated with the presence of SAM.

Hypertensive patients who have had an inferior myocardial infarction often mimic the ASH pattern of HCM. In this setting, the septal/posterior wall ratio may exceed 1.5 simply because the septum is mildly hypertrophied and the posterior wall is thinned as a result of the prior infarct [9].

The Asymmetric Septal Hypertrophy pattern may occur with or without left ventricle outflow tract (LVOTO).

#### Left Ventricle Outflow Tract Obstruction (LVOTO)

The presence of resting obstruction is defined as a peak LVOT gradient >30 mmHg. It has prognostic significance in HCM as a predictor of the risk of sudden cardiac death (SCD) and progression to heart failure [43]. LVOTO arises due to narrowing of the LVOT by septal hypertrophy, anterior displacement of the mitral apparatus and systolic anterior motion (SAM) of the mitral anterior leaflet. The presence of a subaortic membrane and mitral valve abnormalities should be excluded [1].

It has been demonstrated that a steeper LV to aortic root angle is a predictor of LVOTO, irrespective of basal septal thickness [9].

Most patients with HCM do not exhibit significant resting LVOTO but *a dynamic gradient* occurs in *25–30 %* of patients, with the resulting pressure gradient being highly variable and strongly influenced by central blood volume and contractile state [44].

For this reason, all symptomatic patients without evidence of a resting gradient should be investigated for dynamic LVOTO either by Valsalva manoeuvre and exercise test.

Exercise stress echocardiography is recommended in symptomatic patients if bedside manoeuvres fail to induce LVOTO ≥50 mmHg. Pharmacological provocation with Dobutamine is not recommended, as it is not physiological and can be poorly tolerated [45].

The use of glyceryl trinitrate (GTN) is also an option to unmask latent obstruction. Sublingual GTN is administered with the patient supine and evidence of a gradient should be assessed 5–10 min later in a standing position, as the resulting reduction in preload may reveal an intra-ventricular gradient.

#### Systolic Anterior Motion (SAM) of the mitral valve

Systolic anterior motion (SAM) of the mitral valve was first described as a feature of HCM in the late 1960’s, and, although initially thought to be diagnostic of HCM, it has now been showed in many other conditions (including patients with no other evidence of cardiac disease). We know that ∼ 30–60 % of patients with HCM present with SAM and, in 25–50 % of these, left ventricular outflow tract obstruction (LVOTO) is also demonstrated.

Marked systolic anterior motion of mitral valve (with prolonged mitral-septal contact) is more common in patients with diffuse and extensive hypertrophy involving two to four left ventricular segments than in patients with only one hypertrophied segment [8].

The presence of SAM is then not pathognomonic for HCM and may also occur with:

1. other causes of hypertrophy,
2. in hyperdynamic states, or
3. in hypovolaemia (particularly common in dialysis patients) [1].

The haemodynamic consequences of SAM include the prolongation of the ejection time and the reduction of stroke volume. Coaptation of the mitral leaflets may be disrupted resulting in mitral regurgitation.

**Fig. 3:**
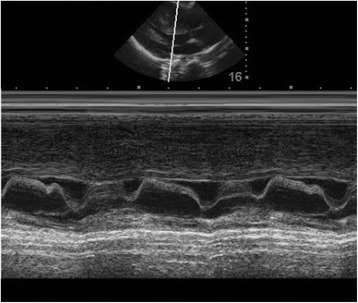
PLAX M-mode of SAM documented by the contact of the anterior mitral valve leaflet/chordae with the septum

The presence of SAM is documented using M-mode echocardiography and is characterized by mid-systolic notching of the aortic valve and contact of the anterior mitral valve leaflet/chordae with the septum. Its severity can be inferred from the duration of leaflet/chordal contact with the septum, being mild if contact occurs for <10 % of systole, and severe if >30 % of systole [46] (Fig. 3).

SAM of the mitral valve in hypertrophic cardiomyopathy (HCM) has generally been explained by a Venturi effect related to septal hypertrophy, causing outflow tract narrowing and high velocities. Patients with HCM, however, also have primary abnormalities of the mitral apparatus, including anterior and inward or central displacement of the papillary muscles, and leaflet elongation. These findings have led to the hypothesis that changes in the mitral apparatus can be a primary cause of SAM by altering the forces acting on the mitral valve and its ability to move in response to them. Despite suggestive observations, however, it has never been prospectively demonstrated that such changes can actually cause SAM [47].

#### Massive septal hypertrophy

It is a rare HCM phenotype characterized by a septal diastolic thickness ≥ 30 mm (Fig. 2B). It is usually associated with a LVOTO but a RVOT obstruction may also occur with the projection of the massively hypertrophied interventricular septum into the right outflow tract (Fig. 2C). This pattern is associated with an higher risk of arrhythmic sudden death [1].

Spirito P [48]. and colleagues have suggested that severe left-ventricular hypertrophy (wall thickness ≥30 mm) alone is sufficient to warrant ICD therapy [49].

Elliot P [28]. found that the excellent survival in the 40 % of patients with a wall thickness of 30 mm or more and no other clinical risk factors shows that a wall thickness of this magnitude cannot by itself be used as justification for implantation of an ICD in patients with hypertrophic cardiomyopathy. Nor does it support the assertion that the absence of massive hypertrophy can be used to reassure patients. This study does, however, suggest that wall thickness may be a useful risk marker when it is included in a broader clinical risk assessment that takes into account other established risk factors such as family history, symptoms, the presence of arrhythmia, and exercise blood pressure responses.

### Asymmetric posterior LV wall hypertrophy

**Fig. 4:**
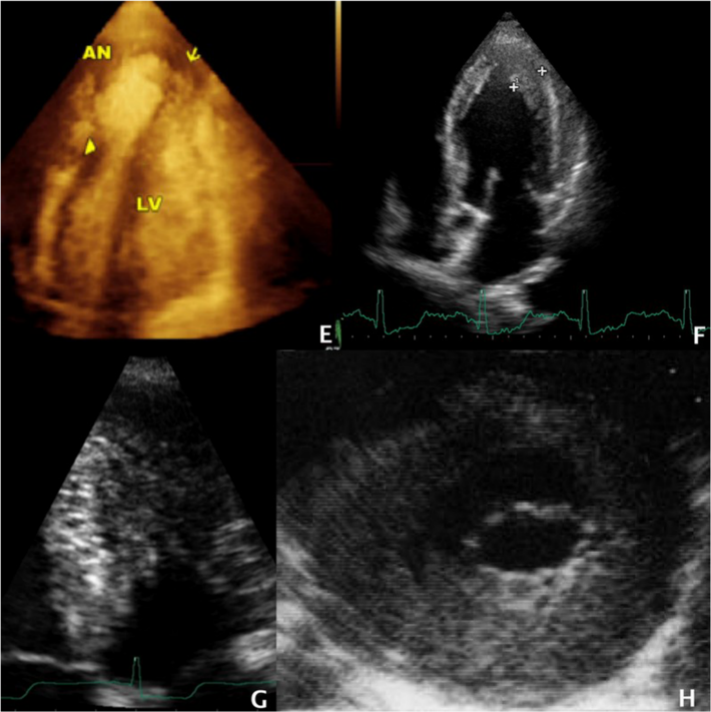
**e** 3DTTE imaging of LV apical aneurysm (from ref. 36). **f** TTE imaging of non massive apical HCM picture. **g** TTE imaging of massive apical HCM characterized by a systolic cavity obliteration. **h** Asymmetric LV posterior wall hypertrophy (from ref. 59)

In 1991, Lewis JF and Maron BJ [50] described a subgroup of patients with hypertrophic cardiomyopathy characterized by an unusual morphologic pattern in which there is marked and often asymmetric thickening of the posterior left ventricular free wall (Fig. 4H). The left ventricular outflow tract is narrowed because of anterior displacement of the mitral valve within the small left ventricular cavity. Systolic anterior motion of the mitral valve is usually present. The clinical profile of these patients included outflow obstruction, severe and early symptoms usually refractory to medical therapy and requiring surgical approach.

### Midventricular Obstructive Hypertrophic Cardiomyopathy (MOHC)

MOCH is a rare phenotype with a prevalence of 1 % of all HCM cases [1].

It is characterized by an atypical intraluminal stenosis of the left ventricle. Hypertrophy is detectable only in the mid portion of the left ventricle and involves the papillary muscles, resulting in a systolic obstruction of the mid-ventricle (Fig. 2D).

This pattern shows smaller LV diastolic volumes and a muscular apposition of the septum and LV free wall able to produce a pressure gradient (PG) [11]. The continuous-wave Doppler echocardiography reveals PG with abnormally high flow velocities across the obstruction. Usually a midventricular PG toward the base occurs in systole whereas a PG toward the apex is detectable in diastole [51]. However there may be a paradoxical jet flow from the apex toward the base during the left ventricular isovolumetric relaxation and the early diastolic filling period and also a jet flow toward the apex during the systole.

Diastolic function is usually severely impaired for this phenotype and septal E/e’ is higher in severely symptomatic patients indicating higher estimated LV filling pressure.

The ‘hourglass’ shaped left ventricle consists of two different chambers: the proximal and the distal chamber. The proximal chamber is an enlarged cavity, with thinned walls and an inferior-basal septum bulging (Fig. 2D). The distal chamber usually is an apical aneurism.

This form is present in the Syed’s classification [42].

#### Left ventricle apical aneurism

LV apical aneurysm may be defined as a discrete thin-walled dyskinetic or akinetic segment of the most distal portion of the chamber with a relatively wide communication to the LV cavity [52]. The incidence of concealed apical aneurysm with mid-ventricular cavity obliteration is approximately 1–2 % of all HCM cases [18]. The echocardiographic assessment of the aneurism should include: size (max length or width), dyskinetic/akinetic pattern, thin rims and transmural (and often more extensive) myocardial scarring identified by late gadolinium enhancement on CMR. Specific complications are more common in association with large or medium rather than with small aneurysms and they consist of: sudden death, LV systolic dysfunction, progressive heart failure symptoms, embolic stroke by LV apical thrombus [16, 17–52].

Diagnostic accuracy for LV apical aneurysm is 57 % for echocardiography (more for medium/large in just 2 dimensions provided by 2D-aneurism), 80 % for echocardiography with the use of a contrast agents (Fig. 4E) and 100 % for CMR [53].

3D-TTE indeed, can provide a more comprehensive assessment of the apical aneurysm as compared to 2D-TTE, which provides at any given time only a thin slice of a structure being studied [17]. With 3D-TTE, the entire extent of the aneurysm can be contained in the 3D dataset so that it could be more fully studied using multiple cross sections at any desired angulation. Measurements in 3 dimensions, including the azimuthal dimension (z axis), allow to assess the volume of the aneurysm, that it is not possible to measure in just 2 dimensions provided by 2D-TTE. This would allow a more accurate monitoring of the progression of the aneurysm over time. A more comprehensive assessment of thrombus is also possible [17] (Fig. 4E).

#### RVOT obstruction in MOHC

HCM should be considered as an extensive process involving both the left and the right sides of the heart. As previously stated, RVOT obstruction may coexist with massive hypertrophy and LVOTO but it could also occasionally be isolated [16, 54–56]. It may be present also in MOHC forms [55].

### Apical HCM

Isolated apical HCM (Helmy’s pattern 4) [39] is a rare variant in the non-Japanese population ranging from 1 % to 2 % [6, 57].

It is a rare phenotype in which the hypertrophy is confined to the LV apex with an apical wall thickness ≥15 mm and a ratio of maximal apical to posterior wall thickness ≥1.5 on 2D-echo [57].

This form is reported in the Syed’s classification [50].

There are some special features of HCM with apex involvement: first, when the apex is involved, ECG evidence of LV hypertrophy is virtually always detectable. In Helmy’s study it was present in 100 % of patients with patterns 3 and 4 [39].

#### Non massive apical HCM

Apical involvement (with a end-diastolic thickness < 30 mm) may be in combination with other LV segments’ hypertrophy (Helmy’s pattern 3 [39]).

This form is generally judged to have a favourable outlook, with a very low risk of developing obstruction or apical aneurysm (Fig. 4F).

Patients usually are asymptomatic and the diagnosis is made following routine ECG [57].

#### Massive apical HCM

The massive hypertrophy of the LV apex is known as ‘Japanese’ phenothype.

It is characterized by a systolic cavity obliteration at TTE assessment [57] (Fig. 4G).

It is associated to the risk of aneurism formation probably because of a micro-vascular myocardial ischemia causing myocardial scarring. In a previous study, 32 % of patients with apical aneurysm had distal hypertrophy alone [52].

### Mild hypertrophy phenotypes

The categories of patients with mild hypertrophy and of patients with non-diagnostic morphological abnormalities (ie. abnormal myocardial strain, systolic anterior motion or elongation of the mitral valve leaflets and abnormal papillary muscles) pose specific and often difficult clinical problems. These features can represent a HCM fenotype that although apparently is a mild form of the disease but in fact it is not without risks.

In 2009, Maron MS et al. [6], using Cardiac Magnetic Resonance (CMR), concluded that patterns of LV hypertrophy are usually not extensive in HCM, involving <50 % of the chamber in about one-half the patients, and are particularly limited in extent in an important minority. Contiguous portions of anterior free wall and septum constituted the predominant region of wall thickening, with implications for clinical diagnosis [6].

Coppini R et al. [58] noted several differences in the echocardiographic evaluation between thick and thin-filament mutation forms.

Patients with thin–filament mutations had lesser maximal wall thickness values than thick filament and more often show atypically distributed hypertrophy including concentric and apical patterns with the lower prevalence of resting LVOT obstruction. Thin–filament patients have smaller LV mass index and lower LVEF(%).

Patients with thick-filament HCM presented a classic asymmetric LVH involving the basal septum and anterior wall.

Coppini R [58] showed a correlation between thin-filament gene mutation and clinical phenotype/outcome. In adult HCM patients, thin-filament mutations are associated with increased risk of LV disfunction and heart failure compared with thick-filament disease, whereas arrhytmic risk in both is comparable. Triphasic LV filling is particularly common in thin-filament HCM, reflecting profound diastolic dysfunction.

Levine RA [47] demonstrated that that primary structural changes in the mitral valve and its supporting structures and their relation to the outflow tract, as observed in patients with HCM, can cause SAM in the absence of significant septal hypertrophy.

SAM appears to be determined by two factors: the ability of the leaflets to move anteriorly (papillary muscle displacement causing slack and increased residual leaflet length) and their interposition into the outflow stream by anterior displacement, determining the direction of this motion. Leaflet slack can permit prolapse (excess superior and posterior motion) or SAM (excess superior and anterior motion), depending on how the papillary muscles shift the orientation of the leaflets relative to the outflow. All these findings can be assessed by echocardiography [47].

## The impact of different echo-patterns of hypertrophy on clinical course and prognosis

The main questions of this article are the following: 1) why is it important to know the type of hypertrophy? 2) What is the clinical impact or prognostic implication of different types of hypertrophy?

The impact of different patterns of hypertrophy on clinical course/prognosis of HCM patients has generated increased interest.

We reported some cases in which the echocardiographic pattern may impact significantly on the clinical course and prognosis.

1. The clinical impact and prognosis of the *ASH* is related to LVOTO development, especially when a SAM of mitral valve leaflets is present. The LVOTO increases the risk of evolution to the *end stage echo-pattern* [59] when small cavity regresses and evolves into a picture similar to that of a dilated cardiomyopathy, with decreased LV systolic function and a dilated left ventricle. Interventricular and intraventricular delays are commonly present in patients with ASH-HCM, despite the absence of conduction abnormalities on the electrocardiogram, and appear to correlate to the degree of septal LVH and the presence of LV outflow obstruction. A study of 123 patients with HCM found that an intraventricular delay ≥45 ms predicted an increased risk for ventricular tachyarrhythmias an sudden cardiac death at 5-years follow-up (85.5 % sensitivity; 90.4 % specificity; positive predictive value: 66.9 %; negative predictive value: 96.7 %; test accuracy: 88.8 %) [58].
  The treatment of this form is aimed to relieve the subaortic PG, decreasing symptoms and improving prognosis.
2. The massive hypertrophy pattern, with a wall thickness ≥30 mm, may be associated with an higher risk of sudden death when it is considered together with other risk factors [48, 28]. Recently, O’Mahony C proposed a novel clinical risk prediction model for sudden cardiac death in hypertrophic cardiomyopathy, including the magnitude of hypertrophy [38].
3. The clinical impact of *asymmetrical LV posterior wall hypertrophy* is related to outflow obstruction often producing severe and early symptoms usually refractory to medical therapy and requiring surgical approach [50].
4. The clinical impact and prognosis of *MOHC* form is related to the specific complications due to apical aneurysm formation. They are more common in association with large or medium rather than with small aneurysms and consist of: sudden death, LV systolic dysfunction, progressive heart failure symptoms, embolic stroke by LV apical thrombus [16, 17, 51–53].
5. Non massive apical form has a modest clinical impact and a favorable prognosis while the massive form is associated to the risk of aneurism formation [56].
6. Mild and atypically distribuited hypertrophy (usually due to thin-filament mutations) are associated with an increased risk of LV disfunction and heart failure compared with thick-filament disease [44].

## Conclusions

It is very important to know and recognize particular echo-features of each HCM phenotype in order to plan the correct treatment and to improve patients’ quality of life and survival.

Echocardiography is still the principal tool for the diagnosis, prognostic assessment and clinical management of HCM. New techniques, such as 3D-TTE and strains curves analysis, are improving their sensibility and specificity. Two-dimensional strain is a simple, rapid, and reproducible method to early detection of abnormalities in patients with HCM and apparently normal left ventricular systolic function.

In this review-article we demonstrate that echocardiographic pattern of the different phenotypes impacts significantly on the clinical course and prognosis of the disease.

## Abbreviations

HCM, hypertrophic cardiomyopathy; HOCM, hypertrophic obstructive cardiomyopathy; HNCM, hypertrophic non-obstructive cardiomyopathy; LVH, left ventricle hypertrophy; ECG, electrocardiogram; CMR, cardiac magnetic resonance; 2D, two-dimensional; LV, left ventricle; NYHA, New York heart association; LA, left atrium; LVOT, left ventricle outflow tract; SAM, systolic anterior movement; MVO, mid-ventricular obstruction; 3DE, 3-dimensional echocardiography; TDI, tissue doppler imaging; STE, speckle tracking ecocardiography; GLS, global longitudinal strain; EF, ejection fraction; TTE, trans-thoracic echocardiography; TEE, trans-esophageal echocardiography; ESC, European society of cardiology; PW, pulsed wave; LAD, left anterior descending; IRT, isovolumetric relaxation time; DT, deceleration time; RA, right atrium; ASE, American society of echocardiography; LAVi, left atrial volume indexed; ASH, asimmetrical septal hypertrophy; LVOTO, left ventricle outflow tract obstruction; SCD, sudden cardiac death; GTN, glyceryl trinitrate; RVOT, right ventricle outflow tract; ICD, implantable cardioverter defibrillator; MOHC, midventricular obstruction hypertrophic cardiomyopathy; PG, pressure gradient; 3D-TTE, three dimensional trans-thoracic echocardiography; 2D-TTE, two dimensional trans-thoracic echocardiography; 3D, three dimensional; LVEF, left ventricle ejection fraction
